# Straight proximal humeral nails are surrounded by more bone stock in comparison to bent nails in an experimental cadaveric study

**DOI:** 10.1186/1754-9493-8-18

**Published:** 2014-04-22

**Authors:** Christian Max Günther, Peter Ernst Müller, Wolf Mutschler, Christoph Martin Sprecher, Stefan Milz, Volker Braunstein

**Affiliations:** 1 University Hospital of Munich, Department of Trauma Surgery - Campus Innenstadt, Ludwig-Maximilians-University, Nußbaumstr. 20, 80336 Munich, Germany; 2 University Hospital of Munich, Department of Orthopaedic Surgery, Physical Medicine and Rehabilitation - Campus Großhadern, Ludwig-Maximilians-University, Marchioninistr. 15, 81377 Munich, Germany; 3 AO Research Institute Davos, Clavadelerstrasse 8, 7270 Davos, Switzerland; 4 Anatomische Anstalt, Ludwig-Maximilians-University, Pettenkoferstraße 11, 80336 Munich, Germany; 5 Sportsclinic Germany, Ottobrunnerstrasse 55, 81737 Munich, Germany

**Keywords:** Bent vs. straight proximal humeral nails, Bone stock, Reduce risk of implant failure

## Abstract

**Background:**

In the management of proximal humeral fractures intramedullary implants with bent and straight shape of the proximal part of nail are available. Based on data from previous studies on bone distribution in the humeral head, we hypothesized, that higher densities might exist in the bone stock surrounding straight nails in comparison to their angulated counterparts. With a known positive correlation between bone density and mechanical stability, this could indicate potentially higher rigidity of osteosyntheses done with straight implants.

**Methods:**

We performed high resolution peripheral quantitative computed tomographies of the potential straight and bent implant bearing regions of 27 cadaveric proximal humeri. The acquired data were analyzed for differences between straight and bent Volumes of Interest as well as intra- and interindividual bone stock distribution.

**Results:**

For both straight and bent volumes of interest a considerably declining bone mineral density was found in craniocaudal direction. Mean densities of bent volumes were significantly lower in comparison to their corresponding straight counterparts (p < 0.01) Intra-individual comparison yielded high bivariate correlations of the corresponding Volumes of Interest of the right and the left side (p < 0.01).

**Conclusions:**

Based on the volumetric data a statistically relevant biomechanical superiority of straight shaped implants can be assumed. Since we found a rapid decrease of bone density in cranio-caudal direction, intramedullary implants should be anchored as proximally in the subcortical area as possible to minimize the risk of displacement or cutout. The high correlation between the Volumes of Interest of the corresponding right and left sides could aid in preoperative planning when considering an intra- or extramedullary approach.

## Background

Proximal humerus fractures are the second most common fracture of the upper extremity [1] with an incidence ranging age- and sex-dependent between 6 and 440 per 100.000 person-years [2-4] with an exponential increase after the 5th decade and a female predominance of 1.5:1 up to 3:1 [3,4]. 15-40% of these fractures need to be addressed operatively [5,6]. During the process of aging a substantial, systemic loss of bone mass and microstructural integrity occurs, concurring with an increasing fracture risk in general as well as locally in the area of the proximal humerus [7]. Epidemiologic distribution of age and sex in patients with proximal humeral fractures which are typically low energy traumata suggest an at least partially relevant relation to osteoporosis. As early as 1947 Berndt described age and sex dependent differences of bone structure in proximal humeri based on radiologic findings [8]. Barvencik et al. found lowered bone density in the lateral humeral head, which could be an explanation for the high incidence of avulsion fractures of the greater tubercle in postmenopausal women [9]. The presence of osteoporotic bone stock impairs the outcome of osteosyntheses by means of insufficient implant fixation putting the patient at risk for subsequent revision procedures. While bone structure is essential for the stability of the bone-implant-construct [10], bone quality is an often neglected factor in preoperative planning. Good correlation between bone density and the mechanical stability of cancellous bone is well documented in different regions of the human and bovine skeleton [11-15]. Along with plates, the most commonly used implants in the management of proximal humerus fractures are intramedullary nails. These come in two variations: straight nails, which are inserted at the apex of the humeral head, offering the significant advantage of passing through the muscular portion of the supraspinatus tendon and thus doing less harm to the rotator cuff. On the other hand, insertion can be difficult since the direct insertion path is blocked by the acromion necessitating manipulation of the fracture zone. This lead to the development of proximally angulated nails, which facilitate insertion, but bear the risk of furtherly displacing fractures running through the greater tubercle or even causing iatrogenic fractures. However, for both implants one of the most important aspects is the primary stability of the osteosynthesis preventing implant failure, especially in the osteoprotic bone. While both implant designs are readily available and are widely used, it is unclear which of them offers higher stability. Since the screws and the distal part of the central rods are the common denominator of both designs, the main factor influencing anchoring stability should be the bone stock surrounding the proximal portion of the nails. Though the aforementioned studies already provide valuable data, they involve an arbitrary selection of the analyzed regions with little comparability to the realistic circumstances of intramedullary nails. In addition, the previously used radiologic techniques were either of a two-dimensional nature or lacked sufficient resolution rendering a detailed analysis of the regions or volumes of interest impossible. Thus the purpose of the current study was to gather high resolution quantitative data (bone volume vs. total volume, BV/TV) of the cancellous bone surrounding the cranial parts of proximal humeral nails, allowing estimates towards the mechanical properties of each design. These data should lead to a recommendation for straight or bent implants, contributing to the effort of achieving high surgical success rates. Based on previous studies we hypothesized that the bone stock decreases in craniocaudal direction. Also we expected the surroundings of the straight nails to have higher densities in comparison to the bent nails due to a known area of lower density and biomechanical stability around the greater tuberosity [16].

## Methods

Twenty-seven humeral specimens (complete humeri, 14 male specimens, 13 female specimens, 13 corresponding pairs) were harvested from 14 post-mortem donors of our anatomical institute, immediately frozen and stored at −20°C. The overall mean age was 71.1 (SD ± 12.9; range 42–98) years. While the mean age of the male donors was 62.8 (SD ± 10.7; range 42–74) years, the female donors mean age was 78.9 (SD ± 9.6; range 68–98) years. Prior to the radiologic analyses the specimens were thawed for 24 hours at 4°C. No humeri with a donor history of cancer, diabetes, bone metabolism altering medication, severe liver or kidney disease, prolonged immobilization, signs of recent or earlier fractures or operative interventions as well as indications of malignancy were included. This trial conforms to the Helsinki Declaration and to local legislation. The ethics committee of the University Hospital of Munich (LMU) approved the trial.

### HR-pQCT analysis

The radiologic analysis was done using the high resolution peripheral quantitative computed tomography (HR-pQCT) imaging system (XtremeCT, Scanco Medical, Brüttisellen, Switzerland) which is equipped with a 70 μm focal spot. The x-ray tube was operated at 60 kVp and 900 μA. The integration time was set to 300 milliseconds. Two-dimensional CT images were acquired, and reformats in 1536 × 1536 pixel matrices from 750 projections using a standard convolution-backprojection procedure were performed. Images were stored in 3-dimensional arrays with an isotropic voxel size of 82 μm. For scanning, the proximal humerus was fixed horizontally with the lesser tubercle in a 12-o’clock position. The complete proximal end of the humerus was scanned, resulting in approximately 550 to 600 microtomographic slices, corresponding to a length of 50 mm. All scans, image processing and analysis were performed by the same person using the proprietary software package provided by the scanner manufacturer (Image Processing Language, Scanco Medical, Brüttisellen, Switzerland). A Gaussian filter with a sigma of 0.7 and support of 1 voxel was primarily used to suppress noise.

### Selection of volumes of interest, segmentation, bone volume calculation

**Figure 1:**
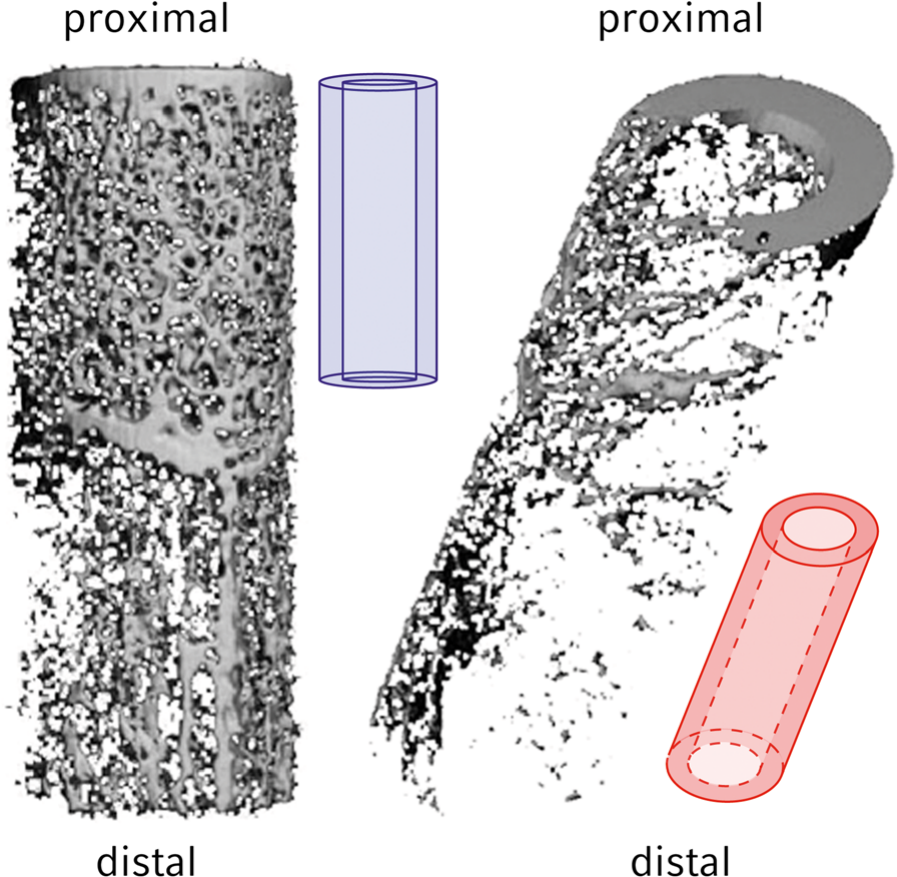
**Volumetric reconstruction of a specimen’s bent and straight regions of interest.** Even macroscopically the difference in bone mineral density is visible.

**Figure 2:**
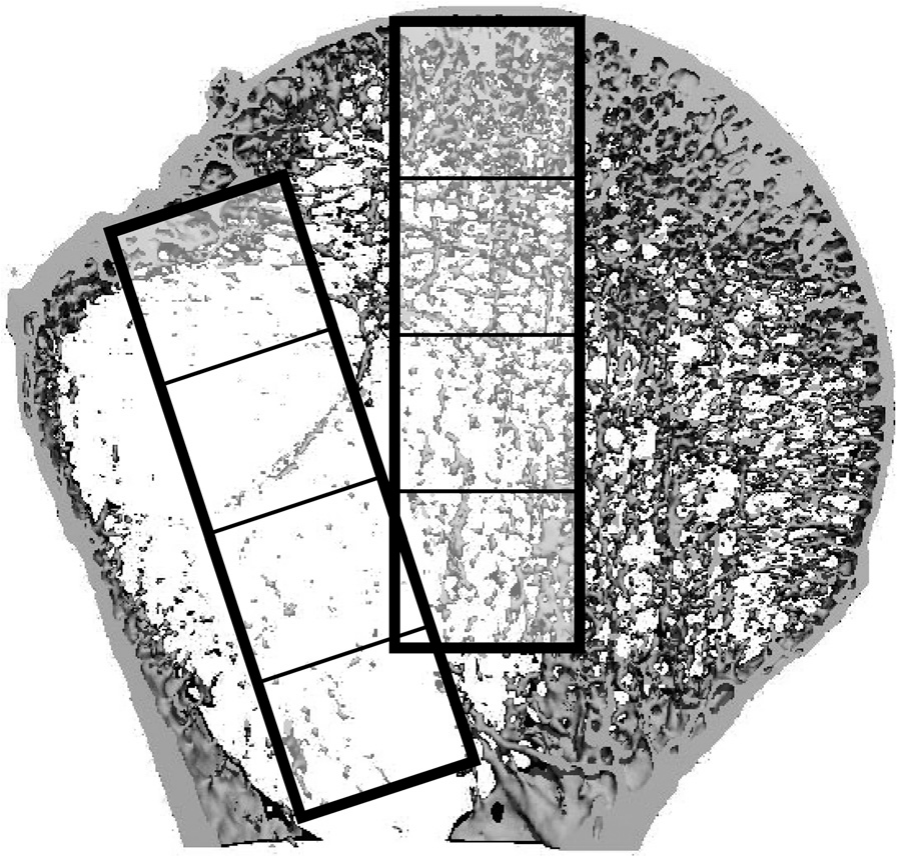
Schematic view of the bent and straight regions of interest (with sub-regions representing tubular quarters of the entire volume) in a frontal plane.

The boundaries of the Volumes of Interest (VOI) was selected on the basis of two of the most commonly used intramedullary proximal humeral nailing systems, the Expert Humeral Nail (Synthes® Inc.) and its bent precursor, the Proximal Humerus Nail, PHN (Synthes® Inc.). The inner diameter of the tubular shaped VOI was set at 9 mm, surrounding the area that has not to be analyzed, since in reality it is filled by the implant, The outer diameter was set at 12 mm, forming a compromise between the area mechanically affected and the discriminatory power between bent and straight volumes. A horizontal line at the most distal point of the glenohumeral cartilage defined the lower border of the humeral head and thus the lower border of the specimen’s VOI to account for different head sizes. Straight VOIs were aligned along to the extended longitudinal humeral shaft axis and oriented transversally at the zenith of the humeral head. Bent VOIs were angulated 5° and positioned according to the implantation specification of the Expert PHN System, which postulates a lateral view positioning in projection of the extended central humeral shaft axis and in anterior-posterior-projection of the bone-cartilage-border (Figures 1 and 2). Since a variable, non-linear distribution of bone mineral density was expected, we further segmented the tubular VOIs into isovolumetric quarters to improve characterization and comparability of the quarters (Figure 2). Those were numbered from proximal to distal with 1 to 4. For binary segmentation, a uniform threshold of 11% of the maximal gray scale value was selected for all samples. This value in the gray value distribution histogram represents the peak characteristic of bone tissue. The segmented VOIs contained only cancellous bone. Separation of cortical from subcortical areas was done manually on inspection using axial views. Bone volume (BV) was calculated using a tetrahedron meshing technique generated with the marching cubes method. The total volume (TV) was defined as the complete volume of the respective VOIs. From BV and TV, the mean bone volume to total volume (BV/TV) value was calculated.

### Statistical methods

Statistic processing of data was done using SPSS Statistics (Version 17.0.0, SPSS Inc., Chicago, IL). Normal distribution was falsified for all groups with the exception of the cortical discs using Shapiro-Wilk- and Kolmogorov-Smirnov-tests with Lilliefors’ correction of significance. Thus, for subsequent analyses, we used non-parametric tests. The comparison of adjacent tubular quarters as well as the comparison of positional corresponding straight vs. bent quarters was done using Mann–Whitney-U-tests. For analysis of right vs. left sides and sex differences we used a Kruskal-Wallis-ANOVA. For side comparison we excluded the single unilateral humerus, reducing the resulting number of specimens of that test to n = 26 (13 pairs).

## Results

**Figure 3:**
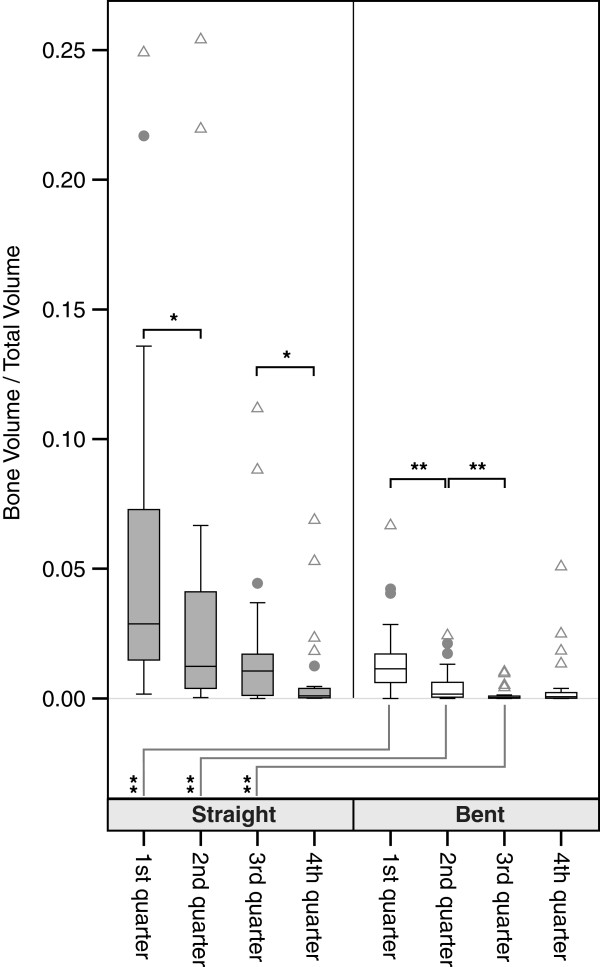
**Boxplots of bone volume/total volume ratio of the overall sample.** Comparisons were done via Mann–Whitney-U-Statistics. Levels of significance: ** p < .01; * p < .05. Outliers are defined as values more distant than 1.5 interquartile ranges from the upper and the lower border of the boxes respectively. Outliers are marked with dots. Extremes are defined as values more distant than 3 interquartile ranges from the upper and the lower border of the boxes respectively. Extremes are marked with triangles.

**Figure 4:**
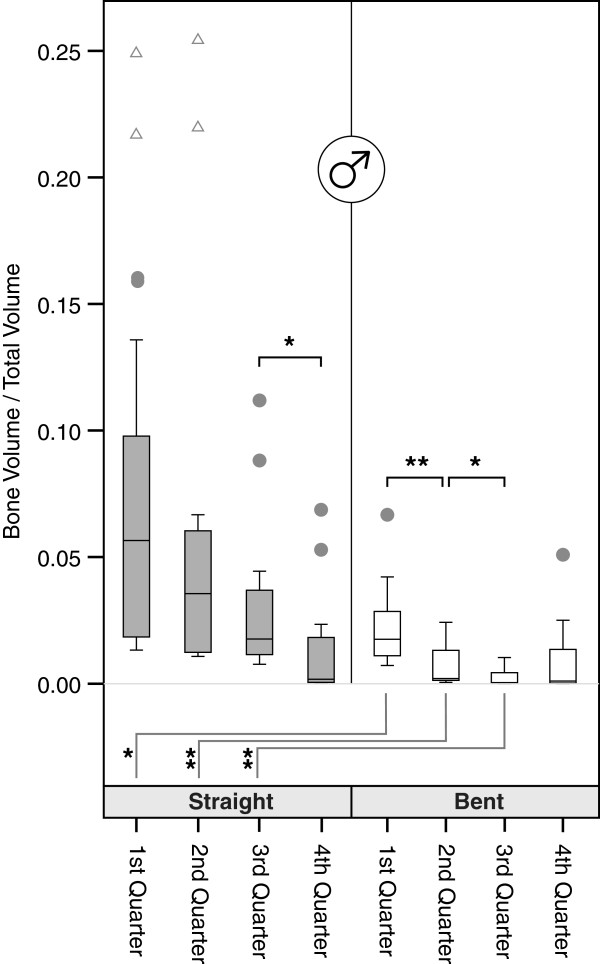
**Volumetric bone mineral densities of the specimens from male donors.** For symbol captions please refer to Figure 3.

**Figure 5:**
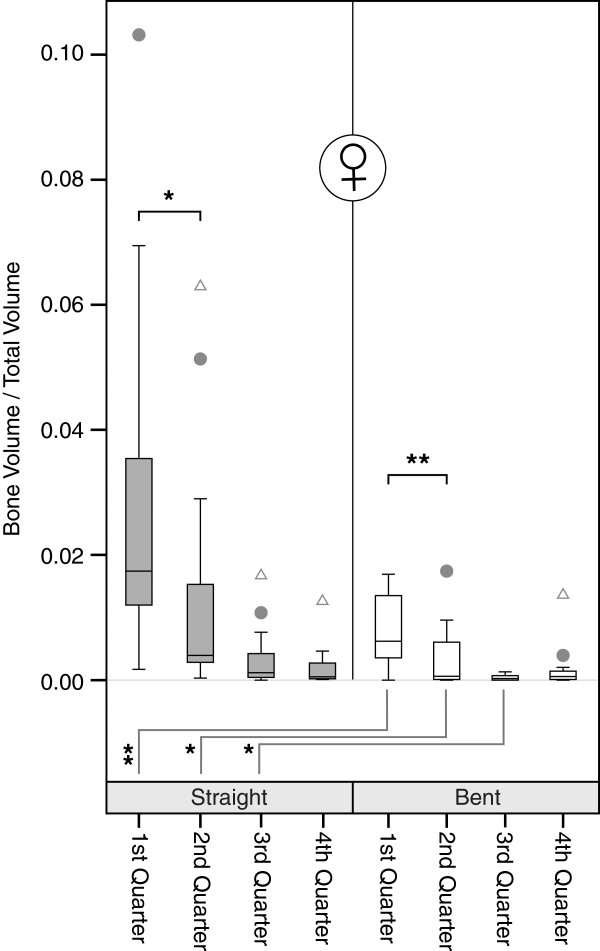
**Volumetric bone mineral densities of the specimens the specimens from female donors.** For symbol captions please refer to Figure 3.

Bent quarters showed significantly lower BV/TV which reach only 11-57% of their corresponding straight counterparts. The differences are significant on a 0.01 level for the quarters, except the most distal quarter. Similarly, looking at the male and female specimens separately significant differences of varying levels exist for the upper 3 quarters (Figures 3, 4 and 5). While cancellous BV/TV show the highest absolute values, subcortically a rapid decline can be seen moving in cranio-caudal direction. The most distal quarter shows a slight increase in density which was caused by elevated BV/TV levels of the most distal bent quarters. At closer inspection of the data, this elevation is caused by two outliers representing a 42-year old male and a 76-year old woman in the group of the bent quarters. Looking at the straight volumes of the whole sample, differences between the first and the second as well as the third and the fourth adjacent quarters exist (p < 0.05). The bent VOI segments differ from first to second as well as second to third (p < 0.01). No significant differences between right and left humeri were present (p > 0.05). We even found good to very high bivariate correlations between both sides, all of which were significant, with the exception of the proximal bent quarter. The lower correlation values in this region result from a weak correlation in women.

## Discussion

One of the foremost findings is the significantly lower bone volume fraction of the bent tubular quarters which reach only 11-57% of their corresponding straight counterparts. Though to the best of our knowledge no comparable study exists for the humeral head in terms of resolution and specificity of the volumes of interest, our results in general compare favorably to the literature [9,17,18]. With a strong correlation between radiologic bone densities and mechanical properties of cancellous bone documented in the literature [19], these findings could be interpreted as an indication of mechanical superiority of straight implants. Another considerable advantage of straight nails is their more medially located entry point, allowing them to penetrate though the muscular, well vascularized portion of supraspinatus theoretically reducing the rates of shoulder pain. Also, there have been reports about iatrogenic extension of fracture zones and generation of avulsion fractures of the greater tubercle in the insertion area of bent intramedullary systems [16,20,21] as well as the risk of further dislocation in the process of bent nail insertion [20,22]. The intraregional analysis revealed a rapid decline in bone mineral density moving from cranial to caudal regions. While the decrease is continuous for the straight volumes, the most distal bent quarter shows a slight increase for both sexes. Though this could be attributed to outliers, there is also the possibility of an interference of the bent distal volumes with a persisting epiphyseal growth plate scar [23] which consists of more dense bone tissue than the rest of the humeral head. The culmination of bone density in subcortical areas puts emphasis on correct positioning of an intramedullary nail. While too proximal positioning bears the risk of irritation or even rupture of the rotator cuff, placing the implant too low could cause disadvantages regarding the resulting stability of the osteosynthesis [24-26]. As we expected, we encountered significantly higher densities for male donors in comparison to females for both bent and straight designs looking at all four subvolumes together. Separate analyses of individual subvolumes yielded significance for the proximal three quarters of the straight, but only the most proximal of the bent VOIs, possibly caused by very low absolute densities in that area. Through greater diameters of the proximal implant part, the bone-metal interface area could be increased, allowing for better distribution of transmitted forces and thus better stability of the osteosynthesis itself. On the other hand bigger implants reduce the biologic fraction of the construct, increasing the risk for avascular necrosis and infection. Preoperatively the surgeon has to choose an adequate implant based on the fracture type and quality of bone. Since the ipsilateral side is compromised in structure and there is no way to reliably measure the local bone density the surgeon has to resort to a comparable area. Total VOIs show high correlation regarding sides for both bent and straight designs (see Table 1), suggesting one would very well be able measure the uninjured side by means of pQCT, facilitating the choice between an intra- vs. an extramedullary approach. Of course, several other factors such as variations in different manufacurers’ implant design and the fact, that modern implants offer an increasing number of customizable locking options, have an impact on the biomechanical stability of the osteosynthesis. Quantity, type and placement of the locking screws or the presence of a locking blade generate a multitude of scenarios. The present study focuses on the isolated situation around the central core of the implant. Subsequent biomechanical testing of the specimens used in the study could give additional information about the stability of the osteosynthesis and allow for correlation of the BV/TV values and failure loads. Also, finite element modelling based on the HR-pQCT images could be used to simulate the bone – implant interaction, with diverse nails. Previous studies have indicated that higher predictive strength regarding stability of cancellous bone can be obtained taking parameters of trabecular microstructure like direction, girth, quantity and interconnectivity into consideration [27,28]. As a consequence a three dimensional morphometric analysis of the respective parameters using the present data sets should follow. In addition, an even more realistic estimation of the bone implant interface region in humeral heads could be achieved taking the surrounding of the locking screws into the equation.

**Table 1:** Intraindividual correlations between right and left humeri

	**Straight VOIs**	**Bent VOIs**
All VOIs (1st-4th quarter)	0,923**	0,919**
1st quarter	0,885**	0,539
2nd quarter	0,918**	0,631*
3rd quarter	0,953**	0,771**
4th quarter	0,888**	0,825**

Very high intraindividual bivariate correlations (Spearman’s Rho-test) were found between corresponding right and left humeri. The lower values for the first and second bent quarters are based on lower correlations in female humeri. Levels of significance: **p < .01; *p < .05.

In the literature both concordant [2,3] and separate [16] age peaks for the incidence of proximal humerus fractures have been reported. However, interpreting the results of our study, one has to take into account, that the male donors in the sample are considerably younger than the female ones. Also the small group sizes of 14 male and 13 female donors should be considered. For statistical workup of the volumes of interest an arbitrary segmentation of the entire VOI is necessary. Increasing the number of sub-VOIs can provide a better understanding of the local distribution of trabecular bone. However time and effort consumed by the analysis increase along with the overall complexity of a higher number of subvolumes. While the resolution of the HR-pQCT scanner is one of the best available today, the overall accuracy of the segmentation is limited by its absolute spatial resolution. Since information on handedness and individual activities of daily life was not available for the specimens used in this study, we cannot rule out possible influence of these parameters. Though a direct correlation exists between radiologic features, bone mineral density and mechanical stability of a osteosynthesis construct, it would be premature to disregard other factors such as general condition of the patient, comorbidities, medication, preexisting level of activity, fracture morphology, quality of reduction, soft tissue damage, form and intensity of forces acting on the osteosynthesis and postoperative rehabilitation regime.

## Conclusions

In conclusion, the present study demonstrates bone density distributions in the human humeral head which highlight regional differences in material quantity. These differences in turn suggest that the anchoring stability of proximal humeral nails may be significantly influenced by the shape of the implant. Isolating the bone stock around the nails, our data suggest that implants with straight design might offer higher biomechanical stability in comparison with bent versions. Since we found the highest BV/TV values for both straight and angled VOIs subcortically with a rapid decrease in craniocaudal direction, intramedullary implants, regardless of the design, should be anchored as proximally as possible to minimize risk of osteosynthesis failure. We found high correlations between VOIs of corresponding right and left sides, which could aid in preoperative planning when considering an intra- or extramedullary approach. An analysis of the local 3-dimensional trabecular structures could further aid to optimize implant design and positioning as well as predicting the risk of osteosynthesis failure.

## Abbreviations

BV: Bone volume; TV: Total volume; QCT: Quantitative computed tomography; pQCT: Peripheral quantiativ computed tomography; VOI: Volume of interest; ANOVA: Analysis of variance; SPSS: Statistical package for the social sciences.

## Competing interests

The authors declare that they have no competing interests related to any part of the trial.

## Authors’ contributions

CG participated in the design of the study, performed the statistical analysis and interpretation of the data and drafted the manuscript. VB conceived of, designed and coordinated the study, participated in the statistical analysis and interpretation of the data and helped to draft the manuscript. PM participated in the interpretation of the data and helped to draft the manuscript. WM conceived of and participated in the design of the study and helped to prepare the manuscript. CS participated in the design of the study, the acquisition of the data and helped to draft the manuscript. All authors read and approved the final manuscript.
